# Headspace Solid-Phase Micro-Extraction Method Optimization and Evaluation for the Volatile Compound Extraction of Bronchoalveolar Lung Lavage Fluid Samples

**DOI:** 10.3390/separations11010027

**Published:** 2024-01-11

**Authors:** Antao Gao, Nina Nouri, Keisean Stevenson, Edith T. Zemanick, Jerry A. Nick, Jane E. Hill

**Affiliations:** 1 Department of Chemical and Biological Engineering, University of British Columbia, Vancouver, BC V6T 1Z4, Canada; 2 Department of Pediatrics, University of Colorado Anschutz Medical Campus, Aurora, CO 80045, USA; 3 Department of Medicine, National Jewish Health, Denver, CO 80206, USA

**Keywords:** HS-SPME, method optimization, GC×GC, bronchoalveolar lung lavage fluid

## Abstract

Headspace solid-phase micro-extraction (HS-SPME) is a prevalent technique in metabolomics and volatolomics research. However, the performance of HS-SPME can vary considerably depending on the sample matrix. As a result, fine-tuning the parameters for each specific sample matrix is crucial to maximize extraction efficacy. In this context, we conducted comprehensive HS-SPME optimization for bronchoalveolar lavage fluid (BALF) samples using two-dimensional gas chromatography with time-of-flight mass spectrometry (GC×GC-ToFMS). Our exploration spanned several HS-SPME parameters, including vial size, dilution factor, extraction time, extraction temperature, and ionic strength. The 10 mL vial size, no sample dilution, extraction time of 50 min, extraction temperature of 45 °C, and 40% salt were identified as the optimized parameters. The optimized method was then evaluated by a pair-wise comparison of ten sets of samples. The results revealed that the optimized method yielded an increase of 340% in total peak area and an increase of 80% in total peak number. Moreover, enhancements were observed across nine major chemical classes in both peak area and number. Notably, the optimized method also doubled the number of volatile compounds consistently detected across BALF samples, from 52 to 108.

## 1. Introduction

Headspace solid-phase microextraction (HS-SPME) is a convenient and solvent-free sample preparation technique that enables the extraction of analytes with minimal matrix interference. This technique works by exposing a coated fiber to the headspace of the sample of interest and allowing the volatile compounds to adsorb or absorb onto the fiber coating. The fiber is then thermally desorbed into an analytical instrument, such as gas chromatography–mass spectrometry (GC-MS), for further analysis. HS-SPME is based on the partitioning of the analyte between the fiber coating, the sample headspace, and the sample itself [1,2]. The process is influenced by multiple parameters, e.g., fiber type, incubation time, headspace-to-sample phase volume ratio, extraction time and temperature, agitation type and speed, matrix pH, and ionic strength. Optimizing the most influential and/or most practical parameters for specific sample types is essential for obtaining a higher extraction efficiency [3–6].

HS-SPME has been used in various fields, such as environmental sciences [7,8], food sciences [9,10], and metabolomics [11–13], highlighting its efficacy and advantages in sample preparation. HS-SPME is also considered one of the “greenest” methodologies as the thermal desorption process is solvent-free, thereby enhancing the eco-sustainability of the method [1,14,15]. Volatolomics is a sub-field of metabolomics that studies the thousands of volatile compounds (VCs) emitted from a biological system [16], including the human body [17–20]. These compounds encompass a wide range of chemical classes, including hydrocarbons, aldehydes, ketones, alcohols, nitrogen-containing, sulfur-containing compounds, and others [21]. The analysis of these compounds provides information associated with specific diseases [20], e.g., respiratory diseases [22,23], gastrointestinal diseases [24,25], diabetes [26,27], and lung cancer [28,29]. Moreover, profiling VCs can reveal information on other physiological statuses of an individual, such as diet and lifestyle [30] and environmental exposure [31].

Sample preparation and extraction play a pivotal role in VC profiling. HS-SPME has been widely used for the extraction of VCs from biofluid samples such as blood [32–34], urine [35,36], sputum [37,38], and bronchoalveolar lavage fluid (BALF) [39–42]. With specific regards to BALF, the matrix of interest for our study, HS-SPME has been used in previous studies for the extraction of VCs to diagnose infections in people with cystic fibrosis (CF) [39], evaluate primary graft dysfunction post-lung transplantation [43], and investigate associations with CF lung microbiome [40]. Despite its research importance, we found no published work specifically focusing on the optimization of HS-SPME for extracting VCs from BALF samples.

GC-MS has been utilized for analyzing compounds extracted by HS-SPME. However, conventional GC-MS can cause co-elution of compounds from complex matrices. Therefore, our study employed a comprehensive two-dimensional gas chromatography (GC×GC) coupled with a time-of-flight mass spectrometry (ToF MS) for VC analysis. GC×GC has been an important technique to couple with HS-SPME, which significantly enhances the sensitivity and selectivity compared to the conventional GC-MS system, providing three- to ten-fold more peaks [44–46].

For advancements in volatolomics research, it is important to extract and identify as many features as possible. Hence, here, we aimed to partially optimize the HS-SPME method to maximize both the number and intensity of VCs extracted from BALF samples. This optimization work will aid in the discovery of volatile biomarkers with potential diagnostic capability.

## 2. Materials and Methods

### 2.1. Study Subjects and Specimen Collection

The BALF samples were originally collected from subjects between two months and 50 years old, all of whom had a confirmed diagnosis of CF. These samples were obtained from 13 CF centers in the US at the time of BALF sample collection [47], following each site’s standard clinical procedure. The samples were collected via a laryngeal mask airway or endotracheal tube. Once collected and stored at −80 °C, the samples underwent an HS-SPME [39]. The samples were then stored at −80 °C.

### 2.2. Sample Processing

For method optimization, 40 BALF samples were thawed on ice and subsequently homogenized using a glass homogenizer (PYREX^®^ Tenbroeck, Corning Inc., New York, NY, USA) to generate a mixed BALF sample. Approximately 5 mL of a phosphate-buffered saline (PBS) solution was added during sample homogenization to ensure an adequate sample volume for the entire optimization experimental design. Subsequently, half a milliliter of the mixed sample was transferred to an individual glass headspace vial. The vial was then sealed with a polytetrafluoroethylene/silicone screw cap and stored at −80 °C for further experiments. The vials and caps were pre-baked at 100 °C for 24 h before use to minimize off-gassing.

### 2.3. HS-SPME Method Optimization

A few parameters were determined based on published literature and experience. The 2 cm tri-phase polydimethylsiloxane/carboxen/divinylbenzene (PDMS/CAR/DVB) *d*_*f*_ 50/30 mm fiber (Supelco, Bellefonte, PA, USA) was selected for the experiment. The incubation time was set as 10 min. The thermal desorption temperature and time were set as 270 °C and 5 min to maximize the desorption efficiency and avoid carry-over issues. The agitation speed was set at 250 rpm, as per the previous literature [48].

#### 2.3.1. Optimization of Vial Size, Dilution Factor, Extraction Time, Extraction Temperature, and Salt Concentration

The optimum vial size was selected by analyzing the undiluted homogenized samples at 43 °C for 30 min using the HS-SPME conditions reported in the HS-SPME optimization paper from our group [48]. Three headspace vial sizes were tested: 20 mL, 10 mL, and 2 mL vials. Due to their height limitation, the 1 cm fiber was employed in the 2 mL vials, while the 10 mL and 20 mL vials utilized a 2 cm fiber. Three replicates were analyzed for each vial size.

The effect of the dilution factor was evaluated by comparing non-diluted samples with the samples diluted (1:2% *v*/*v*) using a PBS solution. The extraction was performed at 43 °C for 30 min, using a 10 mL headspace vial. Three replicates were analyzed for each dilution factor.

A two-variable (k = 2, α = 1.414, block = 2) inscribed rotatable central composite experimental design (CCD) was used to optimize the extraction time and temperature. The extraction time was tested between 20 and 50 min. The extraction temperature was tested within a range of 37 °C to 50 °C. The experiment order was produced by Minitab version 21.1.1 (Minitab, LLC), including nine different sampling conditions (Table 1). Six replicates of the center point run were performed and used to evaluate the precision of the method.

The salt addition effect was assessed at three levels: 0%, 20%, and 40% (% *w*/*v*) NaCl. All the other extraction steps were performed at the optimum condition determined in the previous steps. Three replicates were analyzed for each salt concentration level.

#### 2.3.2. HS-SPME Method Evaluation

To evaluate the effectiveness of our method, we selected 30 BALF samples collected from 30 different subjects. Using samples from various subjects aims to determine whether the optimized method remains effective for samples with diverse biological contexts. These samples were divided into 10 sets, with each set comprising three samples. In each set, the three samples were mixed and homogenized following the same approach described above. The homogenized mixture was then divided into two equal parts and transferred to two individual vials, with each containing 0.5 mL of the mixed sample. The two equally split samples were analyzed using both pre-optimization and optimized methods.

The pre-optimization method used a 20 mL headspace vial with a 2 cm fiber. The sample was 1:2 diluted with a PBS buffer, and no salt was added to the sample. The optimized method used a 10 mL headspace vial with a 2 cm fiber. The sample was not diluted, and 40% (% *w*/*v*) NaCl was added to the sample.

### 2.4. Sample Instrumental Analysis

The samples were thawed at 4 °C on ice for 1 h and subsequently placed in the cooled sample tray. The sample tray was maintained at 4 °C during the sample instrumental analysis. The extraction was carried out using the fully automated HS-SPME mode on the MPS system (Gerstel^®^, Linthicum Heights, MD, USA). Initially, the sample was incubated for 10 min at the extraction temperature with an agitation speed of 250 rpm. Then, the headspace extraction was performed at the determined temperature and time using a PDMS/CAR/DVB fiber. Before sample extraction each day, the fiber was conditioned at 270 °C for 30 min, and then thermally desorbed into the GC×GC to ensure it is cleanliness.

After sample extraction, the fiber was injected into the Pegasus 4D (LECO Corporation^®^, St. Joseph, MI, USA) GC×GC-ToFMS with an Agilent 6890 GC (Agilent Technologies, Santa Clara, CA, USA) through the Septumless Head (SLH) injector and desorbed at 270 °C for 5 min. The primary column was a standard non-polar Rxi-5 MS (Restek Corporation, Bellefonte, PA, USA) (30m × 250 μm; 0.25 μm *d*_*f*_). The second column was a polar Stabilwax (Crossbond^®^ polyethylene glycol) (Restek Corporation, Bellefonte, PA, USA) (1.5 m × 250 μm; 0.25 μm *d*_*f*_). The primary GC oven temperature was initially set at 40 °C, held for 2 min, and increased at 5 °C/min to 230 °C. The temperature offset for the secondary oven was 5 °C relative to the primary oven. A modulation period of 2 s was used. A quad-jet dual-stage thermal modulator was used for modulation. The temperature offset for the modulator was 15 °C relative to the secondary oven. The mass spectrometry was performed at the electron ionization (EI) mode at 70 eV. The mass spectrum was collected within the range of m/z 30–400 at a sampling rate of 200 Hz.

Instrument reproducibility was evaluated by running three Grob programmed test mix injections (Supelco, Bellefonte, PA, USA) before running the actual samples. The percent relative standard deviation (%RSD) of the area counts of each peak in the standard mix was proved to be less than 15%. This test proved the system stability and ensured the instrument is in good condition to analyze samples. One fiber blank was analyzed each day. The peak number should be less than 200, and the maximum peak area should be less than 1 × 106. One alkane standard mix injection (Supelco, Bellefonte, PA, USA) and two Grob programmed test mix injections were included in each batch of sample analysis. Instrument tune and leak tests were performed daily. Carry-over was assessed by desorbing the blank fiber after the Grob programmed test injection. No carry-over was observed after analyzing the actual samples.

### 2.5. Data Processing

Data collection and preliminary data processing were performed using ChromaTOF software, version 4.72 (LECO Corp). For peak finding, a signal-to-noise (S/N) cutoff of 50:1 (with a minimum of three apexing masses) was used in at least one chromatogram and a minimum of 20:1 S/N in all others. The NIST 11 library was used for the initial putative identification of the analytes with a match threshold > 700, as referred to in a previous study [49]. The identified compounds were also given certain chemical classes [50]. Moreover, the chemical identities of a subset of molecules were confirmed by comparing retention indexes with the library reference, using a criterion of difference of less than 30.

### 2.6. Statistical Analysis

The raw data from ChromaTOF software were input into R version 4.2.3 (R Core Team, Vienna, Austria) for statistical analysis. Known artifacts and environmental contaminants [51] (Supplementary Table S1) were first removed from the raw data. Response surface analysis (RSA) was performed using Minitab software (version 21.1.1). The statistical analyses were performed using GraphPad Prism 10 version 10.0.0. The overall performance was evaluated by comparing two variables, including the total peak area after peak deconvolution and the total number of peaks. A one-way ANOVA followed by Turkey’s multiple-comparison test, and Student *t*-tests were employed to test if a statistically significant difference exists among the mean values of different groups. The *p*-value (<0.05) was used as the criteria to ascertain a statistically significant difference. Response surface analysis (RSA) was implemented to interpret the CCD results.

## 3. Results and Discussion

### 3.1. HS-SPME Method Optimization

#### 3.1.1. Optimization of Vial Size

**Figure 1.**
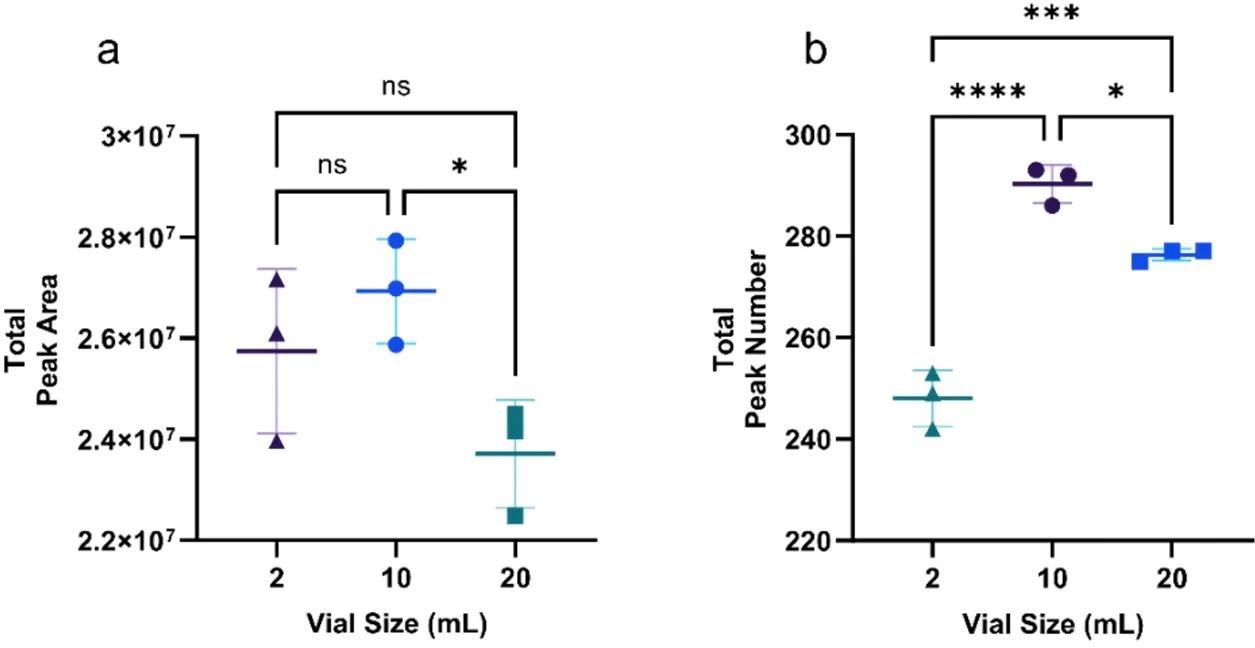
(**a**) The total peak area and (**b**) the total peak number extracted using different vial sizes. The *p*-value was calculated based on the one-way ANOVA test followed by Turkey’s test. * *p*-value < 0.05; *** *p*-value < 0.001; **** *p*-value < 0.0001. The “ns” stands for “not statistically significant”. The shape of the points represents different vial size: triangle—2 mL vial; round dot—10 mL vial; square—20 mL vial.

Vial size can change the headspace-to-sample volume ratio, an important factor that affects the HS-SPME efficiency [52]. We hypothesized that smaller vials would outperform the larger vials in terms of total peak area and total peak number. To evaluate our hypothesis, we tested 2 mL, 10 mL, and 20 mL vials. It should be noted that for 10 mL and 20 mL vials, a 2 cm fiber was used, whereas for 2 mL vial, a 1 cm fiber was used. Although the fiber surface area has a greater impact on the amount of VC extracted, we realized that the headspace-to-sample volume ratio can have a more substantial impact, especially for compounds with a larger partition coefficient between headspace and sample phase (Supplementary, Equation (S1)). The results show that the 10 mL vial surpassed the performance of the other two vial sizes in terms of total peak area and total peak number, showing statistically-significant differences (Figure 1). The 10 mL vial yielded the highest total peak area, with an average value of 2.69 × 10^7^, higher than both the 20 mL (2.37 × 10^7^) and 2 mL (2.57 × 10^7^) vials. Additionally, the 10 mL vial resulted in the highest mean total peak number of 290, surpassing both the 20 mL and 2 mL vials, which extracted an average of 276 and 248 peaks, respectively.

In a system in equilibrium, the amount of volatile compounds extracted on the fiber is negatively correlated with the headspace-to-sample volume phase ratio and positively correlated with the volume of the fiber coating [52,53]. The superior performance of the 10 mL vial, yielding both higher total peak areas and numbers compared to the 20 mL vial, can be attributed to its smaller phase ratio. Interestingly, despite the fact that the 2 mL vial has a more favorable phase ratio than the 10 mL vial, the total peak number extracted by the 10 mL vial was significantly higher than the 2 mL vial, while the difference in total peak area was not statistically significant. We speculate that the reduced fiber surface area might be a decisive factor, resulting in a smaller extracted peak area and a lower total peak number. Moreover, the broader diameter of the 10 mL vial provides an expanded surface area, facilitating the transition of a higher number of compounds into the headspace from the liquid phase [54]. Similar results were reported in an HS-SPME optimization study for urine samples, in which the 10 mL vial yielded a significantly larger mean number of volatile compounds than the 2 mL vial [54].

#### 3.1.2. The Effect of Dilution

**Figure 2.**
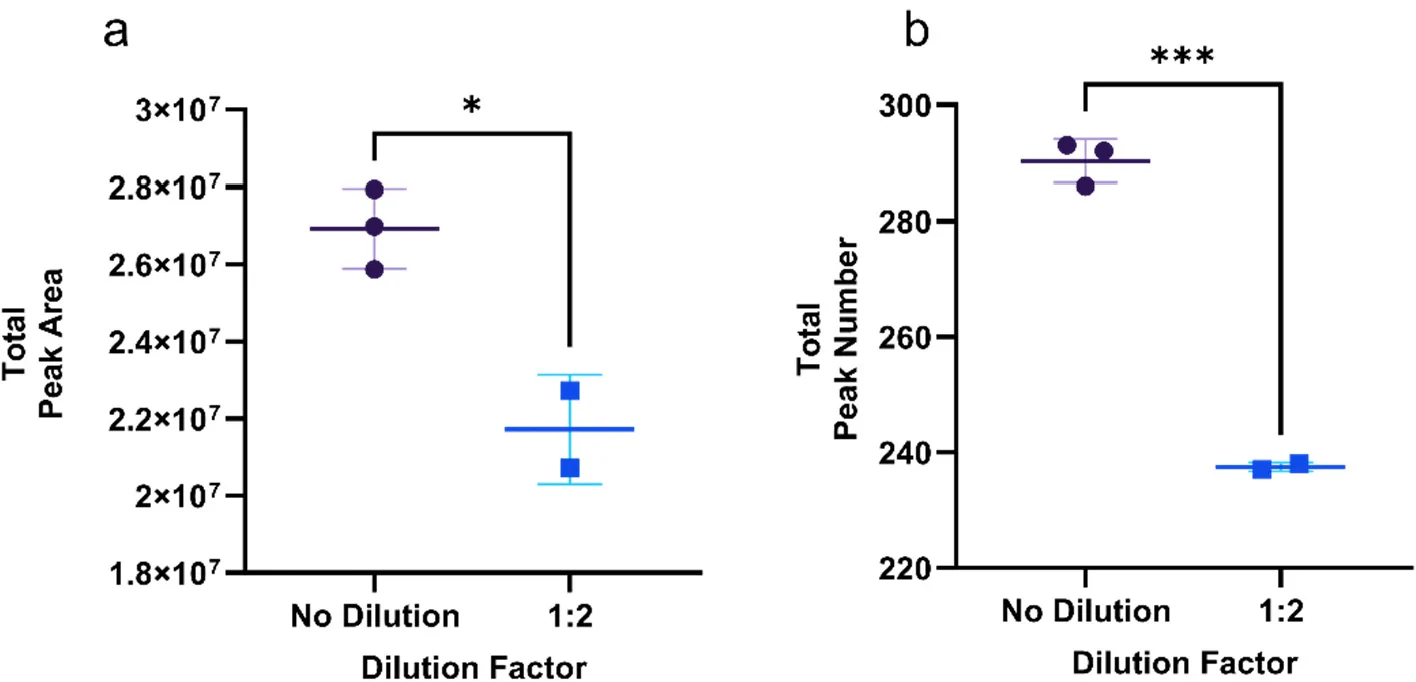
(**a**,**b**) The comparison of the total peak area and total peak number extracted from non-diluted and diluted samples. The *p*-value was calculated based on the student *t*-test. * *p*-value < 0.05; *** *p*-value < 0.001. The shape of the points represents different vial size: round dot—10 mL vial; square—20 mL vial.

Sample dilution is an approach that modifies the sample matrix and influences the HS-SPME efficiency [55]. We hypothesized that undiluted samples would yield higher total peak area and total peak numbers, which is confirmed by our results (Figure 2). Specifically, the non-diluted samples obtained an average of 2.69 × 10^7^ total peak area, whereas diluted samples obtained 2.17 × 10^7^, showing statistically significantly different (*p* value = 0.0167). In addition, the non-diluted samples produced a mean of 290 peaks, which is statistically significantly higher than the 237 peaks obtained from the diluted samples.

It has been reported that for samples with complex and high-concentration matrices, adding diluent may increase the extraction recovery by reducing the interference from matrix components and promoting the shift of analytes to the free form [3]. However, excessively diluting the sample may decrease sensitivity due to a reduced concentration. In two HS-SPME optimization studies [56,57], the authors observed that adding diluent initially increased the extraction performance, but an excessive dilution led to a decrease in extraction efficiency. Another study on plasma samples reported that adding diluent reduced the amount of extracted volatile compounds except for diazepam, which tends to bind strongly with the proteins in plasma [58]. BALF is a saline solution primarily composed of alveolar macrophages and some lymphocytes [59]. We suspect that the BALF is not a complex matrix; hence, the addition of diluent led to a decrease in sensitivity.

#### 3.1.3. Optimization of Extraction Time and Temperature

**Figure 3.**
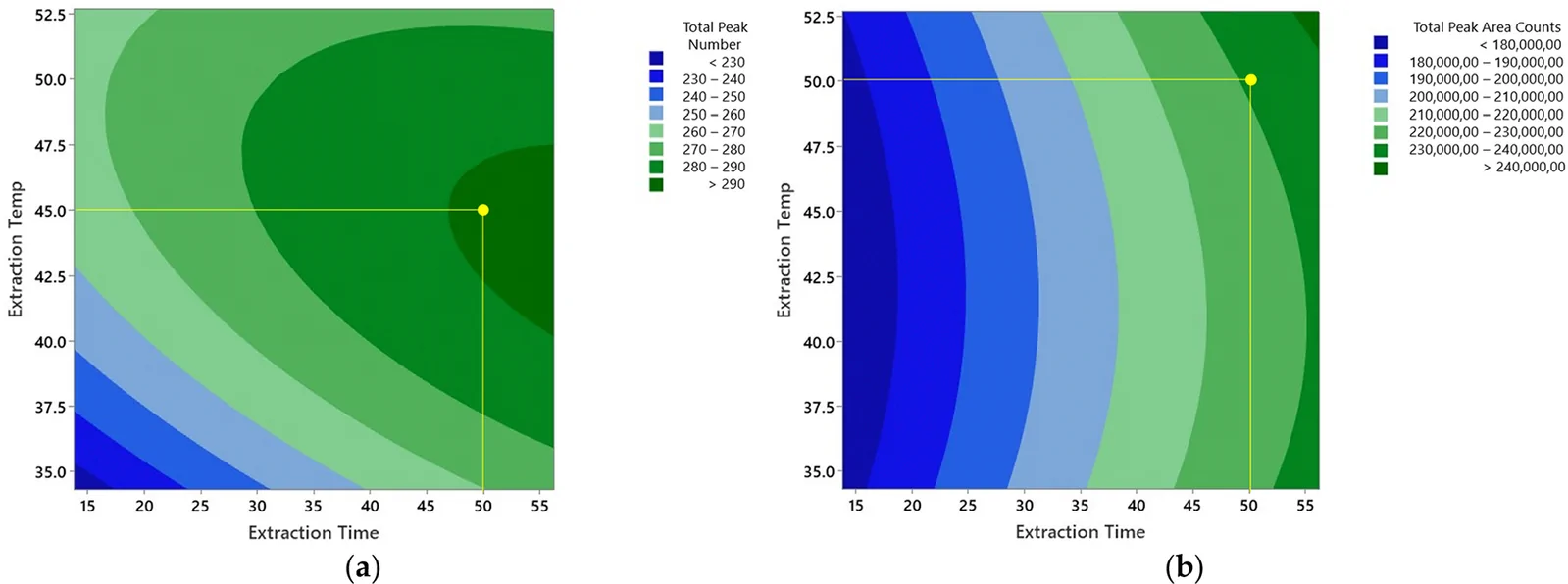
Response surface analysis performed on total peak area and total peak number. (**a**) Total peak area vs. extraction time vs. extraction temperature. (**b**) Total peak number vs. extraction time vs. extraction temperature. Yellow dots represent the optimum point.

We hypothesized that an optimum combination of extraction temperature and extraction time would yield the highest total peak area and total peak number. We observed that the highest total peak area was achieved using an extraction temperature of 50 °C and an extraction time of 50 min. In contrast, the highest total peak number was obtained with the same extraction time but at a slightly lower temperature of 45 °C. The total peak area extracted by these two conditions was 2.25 × 10^7^ and 2.31 × 10^7^, respectively, showing a 2.7% difference. The total peak numbers extracted were 291 and 285, respectively, indicating a 2.1% difference (Figure 3). As these two differences were minor and not statistically different, 45 °C and 50 min were selected as a compromise for high-throughput analysis. The analysis of variance results revealed a lack-of-fit *p*-value of 0.003 for the total peak area and 0.001 for the total peak number, respectively. The output table and Pareto Chart for the CCD are attached in the supplementary file (Figure S1, Table S3). Six replicates of the center point sample showed low relative standard deviation and high reproducibility (Supplementary Figure S3; Table S4).

In a prior HS-SPME optimization work on a cell culture infected with respiratory syncytial virus, the authors evaluated optimization parameters using 12 compounds, targeting a broad spectrum of major chemical classes [48]. Inspired by this approach, we similarly tested our hypothesis by examining 41 compounds (Supplementary, Table S2) from different chemical classes. These compounds meet one of these two criteria [48]: (1) they have a mass spectral similarity score greater than 850, and (2) the difference between the calculated retention index (RI) and the RI in the library is less than 30. After conducting a response surface analysis on these 41 compounds, we observed that none exhibited an optimum plateau in the perspective plot within the specified extraction temperature and time range (Supplementary Figure S1).

Extraction time is a critical parameter of the HS-SPME method, determining whether the system reaches equilibrium across the headspace, sample, and fiber phases. The duration required for a system to reach equilibrium depends on various factors, such as extraction temperature, sample matrix, and the nature of the analytes. For example, the equilibrium in HS-SPME of plasma samples at 37 °C is reached approximately after 90 min [58]. Extraction temperature can influence the equilibrium time, meaning that increasing temperature may shorten the time required for reaching equilibrium [3].

We suspect that our system did not achieve equilibrium under the designated conditions, as indicated by the continuously rising total peak area and total peak number within the given without reaching a maximum value. We believe this could be due to the insufficient extraction time and the relatively low extraction temperature.

#### 3.1.4. The Effect of Salt Addition

**Figure 4.**
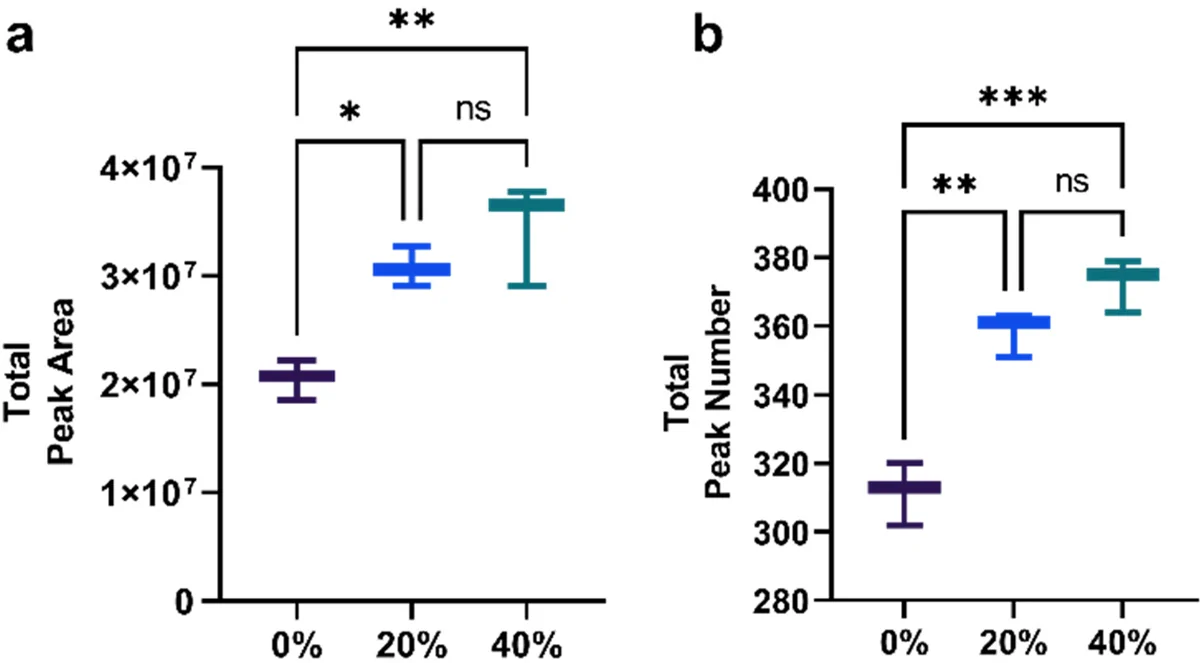
(**a**,**b**) Comparison of total peak area and total peak number of peaks extracted among samples with no salt, 20% salt, and 40% salt; * *p*-value < 0.05; ** *p*-value < 0.01; *** *p*-value < 0.001. The “ns” stands for “not statistically significant”. The color represents different salt concentrations.

We hypothesized that adding salt to the sample would lead to a higher total peak area and total peak numbers. The results show that adding 40% NaCl to the samples significantly enhanced the number of extracted peaks and total peak area, compared to 20% and no salt addition (Figure 4a,b). Specifically, the samples with 40% salt obtained an average total peak area count of 3.44 × 10^7^, whereas samples with 20% salt and samples without salt obtained 3.08 × 10^7^ and 2.05 × 10^7^, respectively. The samples with 40% salt obtained an average total peak number of 373, while those with 20% and no salt obtained an average total peak number of 358 and 312, respectively. For both the total peak area and total peak number, the difference between 40% and no salt was statistically significant.

Salt addition is a common technique to enhance HS-SPME efficiency. Adding salt may increase the activity coefficient of the compounds in the sample, which subsequently leads to an increase in the concentration of the compounds in the headspace [3]. The effect of salt addition depends on salt concentration [53], and it is essential to experimentally evaluate and optimize this parameter. Our finding aligns with other studies, which reported that adding NaCl enhanced HS-SPME extraction efficiency in various sample types, including urine [60], culture media [48], water [61], liquor [56], and cow slurry [62]. We observed that although the average total peak area and peak number were higher in samples with 40% NaCl than those with 20%, the difference was not statistically significant. Similar findings have reported that the salt addition effect is non-linear and tends to reach a plateau at 35% NaCl, approaching the saturation concentration of 40% [32].

**Figure 5.**
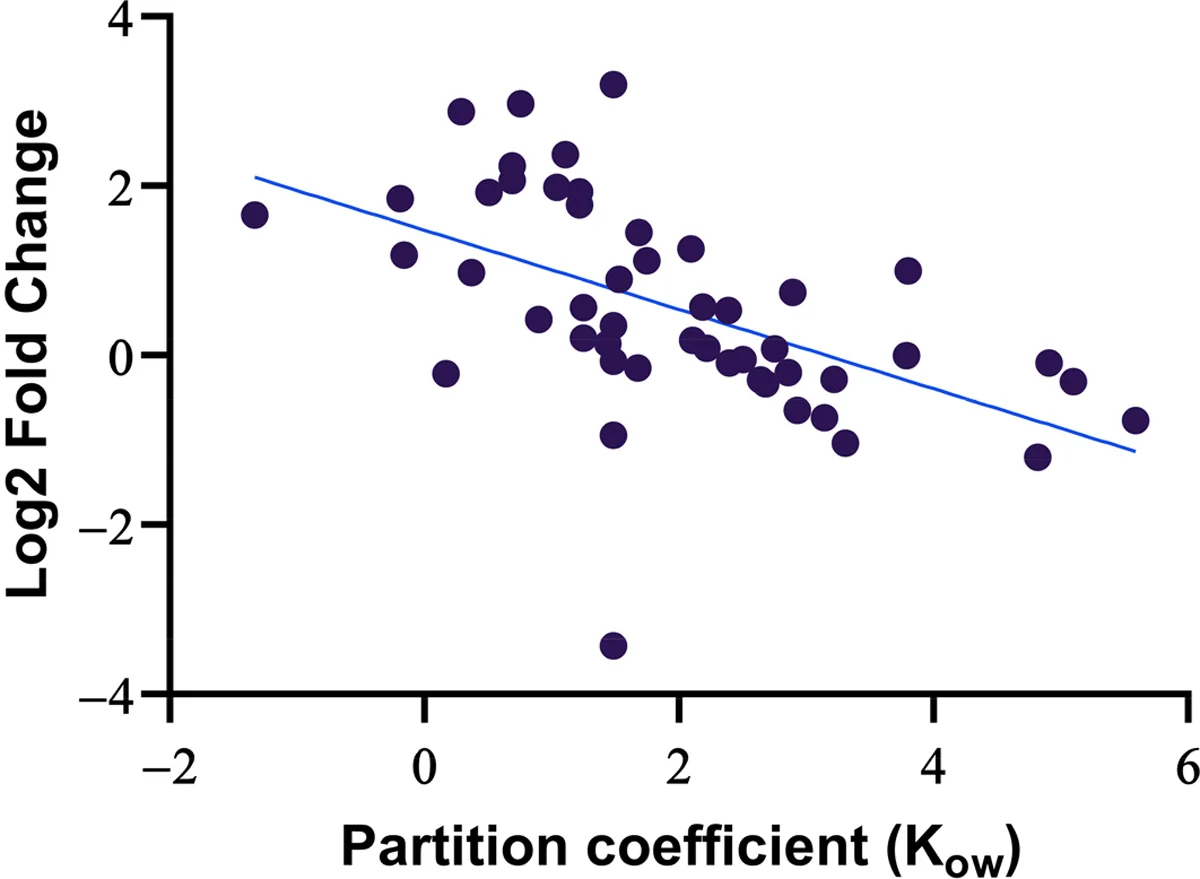
The linear regression between the partition coefficient and Log2 fold change of each of the 46 selected compounds. Pearson’s correlation coefficient = −0.53. The solid line represents the linear regression line with the equation of Y = −0.4679 × X + 1.478.

**Figure 6.**
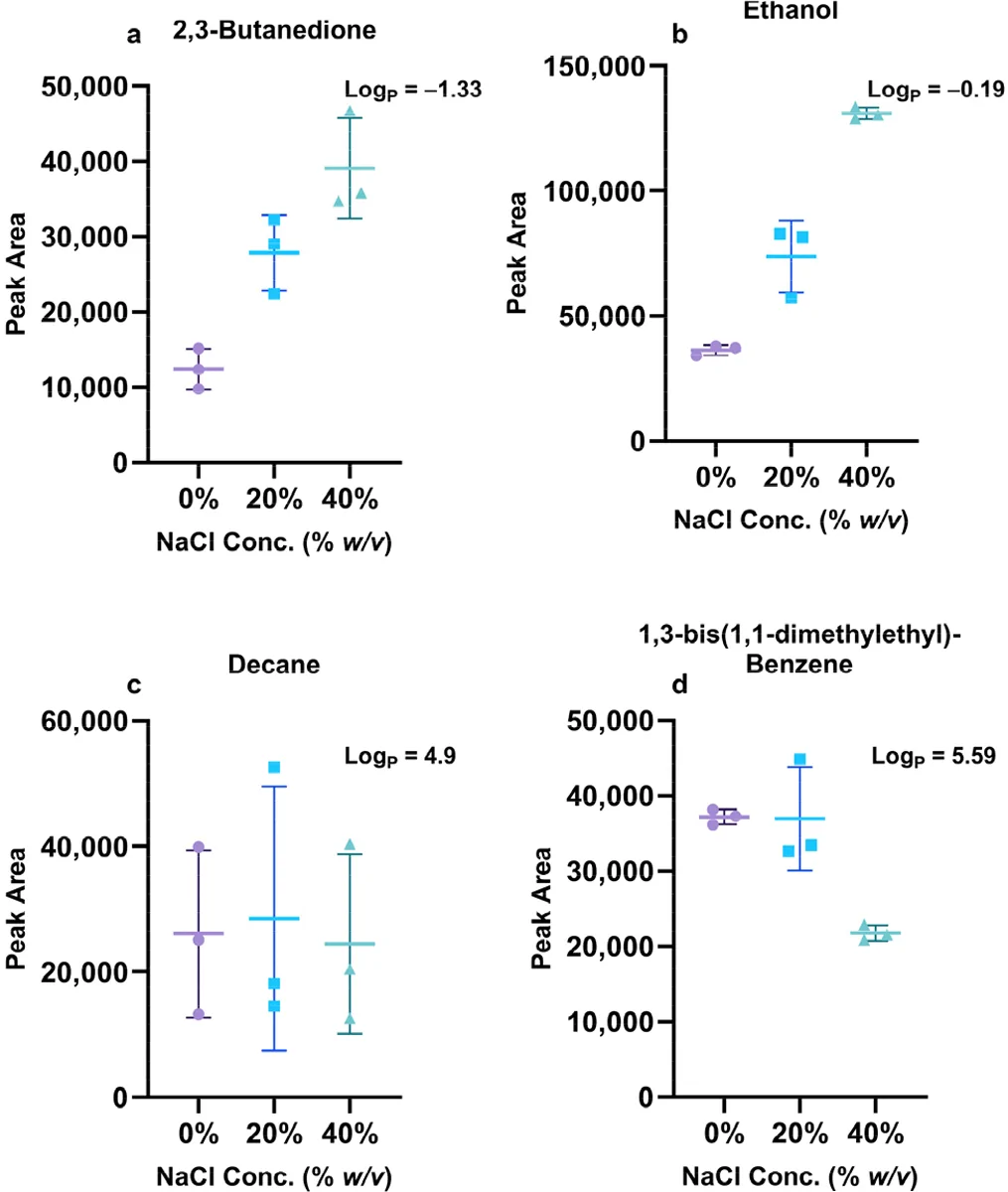
Boxplot of four selected compounds with partition coefficients (*K*_*ow*_) from low to high. (**a**,**b**) show two compounds (2,3-Butanedione and Ethanol) with low *K*_*ow*_ which demonstrate increasing peak area as the salt concentration increases; (**c**,**d**) illustrate two compounds (Decane and 1,3-bis (1,1-dimethyl ethyl)-benzene) with high *K*_*ow*_ which demonstrate no change and a decreasing peak area, respectively, as the salt concentration increase.

It has been mentioned in the theory of HS-SPEM that the salt addition effect increases with increasing compound polarity [53]. Therefore, we also hypothesized that the salt addition would affect polar compounds more than non-polar compounds. To test this hypothesis, we evaluated the salt addition effect on 46 compounds (Supplementary Materials, Table S2), which were selected based on the criteria mentioned in Section 3.1.3. The correlation analysis between the partition coefficient *K*_*ow*_ (octanol/water partition coefficient, used as a substitute for polarity) and the Log2 fold change of each compound showed a negative regression trend (Figure 5). The slope is significantly different from zero (*p*-value < 0.001). Furthermore, we selected the two most polar (2,3-butanedione and ethanol) and the two most non-polar compounds (decane and 1,3-bis (1,1-dimethyl ethyl)-benzene). We studied their variance of relative abundance with increased salt amount. The results showed that for 2,3-butanedione and ethanol, the peak area differences among three NaCl concentration levels were statistically significant, except for 2,3-butanedione between 20% and 40% NaCl. For the two non-polar compounds, while decane exhibited no noticeable variation in peak area across different NaCl concentrations, 1,3-bis(1,1-dimethylethyl)-benzene displayed a decrease with increasing salt concentration (Figure 6).

Based on our observation, we can conclude that adding salt can significantly improve the HS-SPME efficacy. We can also speculate that the salt addition predominantly affects the polar compound rather than the non-polar compound, and the extent of the salting addition effect might correlate with the polarity of the compounds.

In summary, the following parameters were optimized (Table 2).

### 3.2. Method Evaluation

To evaluate the efficacy of the method optimization, we conducted a comparison of the optimized and pre-optimization methods in terms of total peak area and total peak number. The chemical class composition of the extracted compound is important as it may be indicative of the origin of the volatile compounds. Therefore, we examined the peak area and peak number in nine chemical classes. In addition, we examined the number of compounds consistently present in all mixed BALF samples to evaluate if the optimized method can decrease the number of missing values caused by the sample extraction step.

**Figure 7.**
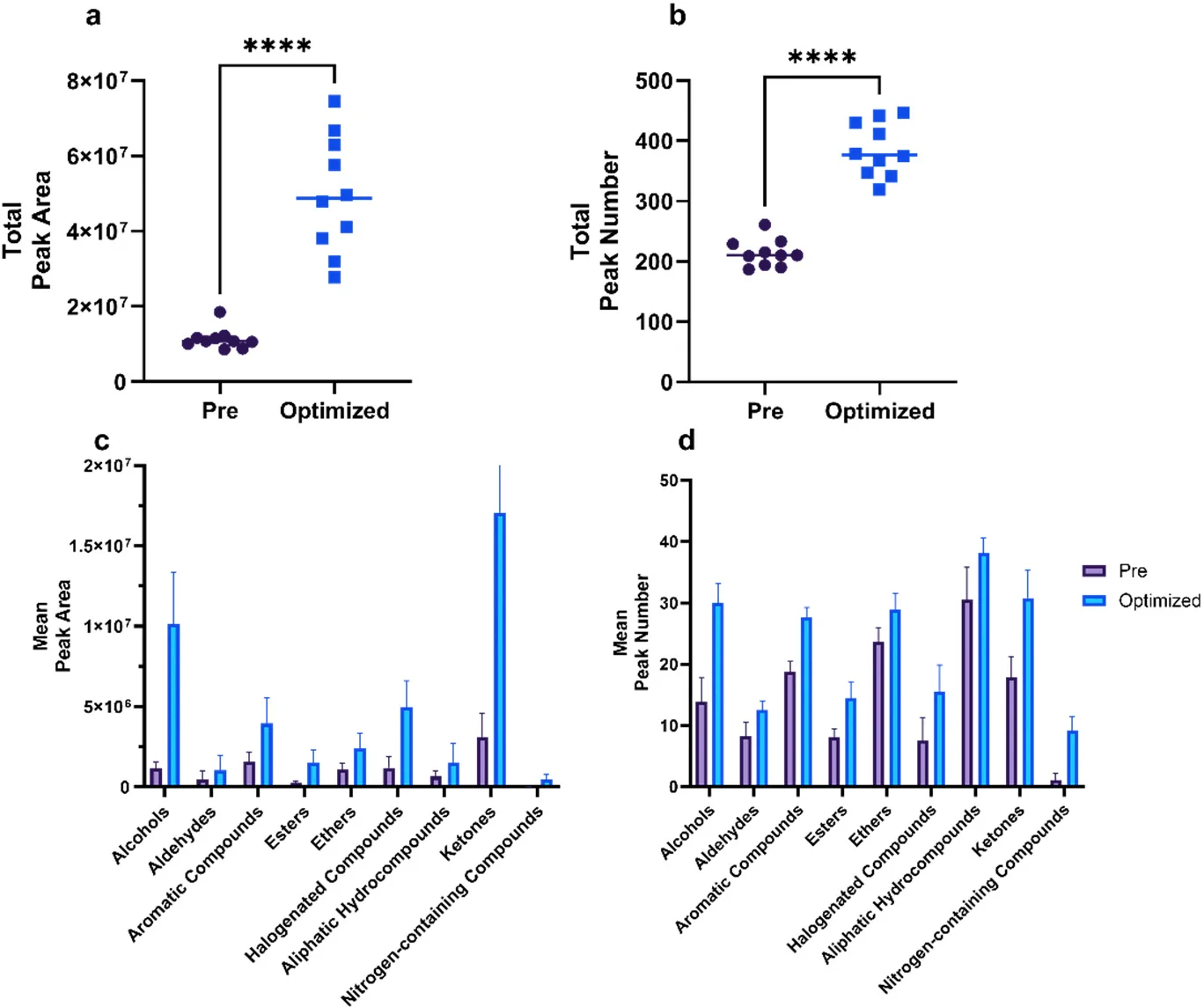
(**a**,**b**) Comparison of the average total peak area and total peak number of 10 sets. (**c**,**d**) Illustration of the variation in the peak area and peak numbers among nine chemical classes. **** *p*-value < 0.0001.

We hypothesized that the optimized HS-SPME method would outperform the pre-optimization method regarding total peak area and peak number. We observed that optimizing the method led to increased extraction efficiency for all 10 pairs of mixed BALF samples. Specifically, the optimized method generated an average total peak area of 4.98 × 10^7^, a 340% increase compared to the pre-optimization method, which was statistically significant (*p*-value < 0.0001) (Figure 7a). Additionally, the optimized method generated an average of 80% more peaks compared to the pre-optimization method, also showing a statistically significant difference (Figure 7b). Our findings show that the optimization of vial size, dilution factor, extraction time, extraction temperature, and salt concentration improves the HS-SPME efficiency regarding total peak area and total peak number.

We also compared the peak area and peak number for nine major chemical classes in the BALF samples extracted using the pre-optimization and optimized methods. We observed that, after optimization, the total peak area increased for nine major chemical classes (Figure 7c). The increase ranged from 112% to 761%, among which alcohols, ketones, and esters have the most significant increase. Specifically, the alcohols increased by 761%, on average, compared to the pre-optimization method; the ketones increased by 447%; and the esters increased by 425%. All nine classes demonstrated statistically significant differences except the aliphatic hydrocarbons. The total peak number also increased for all nine chemical classes (Figure 7d), with the increase ranging from 19% to 116%. The alcohols, halogenated compounds, and esters showed the most notable increase of 116%, 103%, and 79%, respectively. All nine classes demonstrated statistically significant differences. We noticed that the chemical classes with polar functional groups demonstrated a more significant increase. Thus, we may speculate that optimization has a more significant impact on polar compounds.

**Figure 8.**
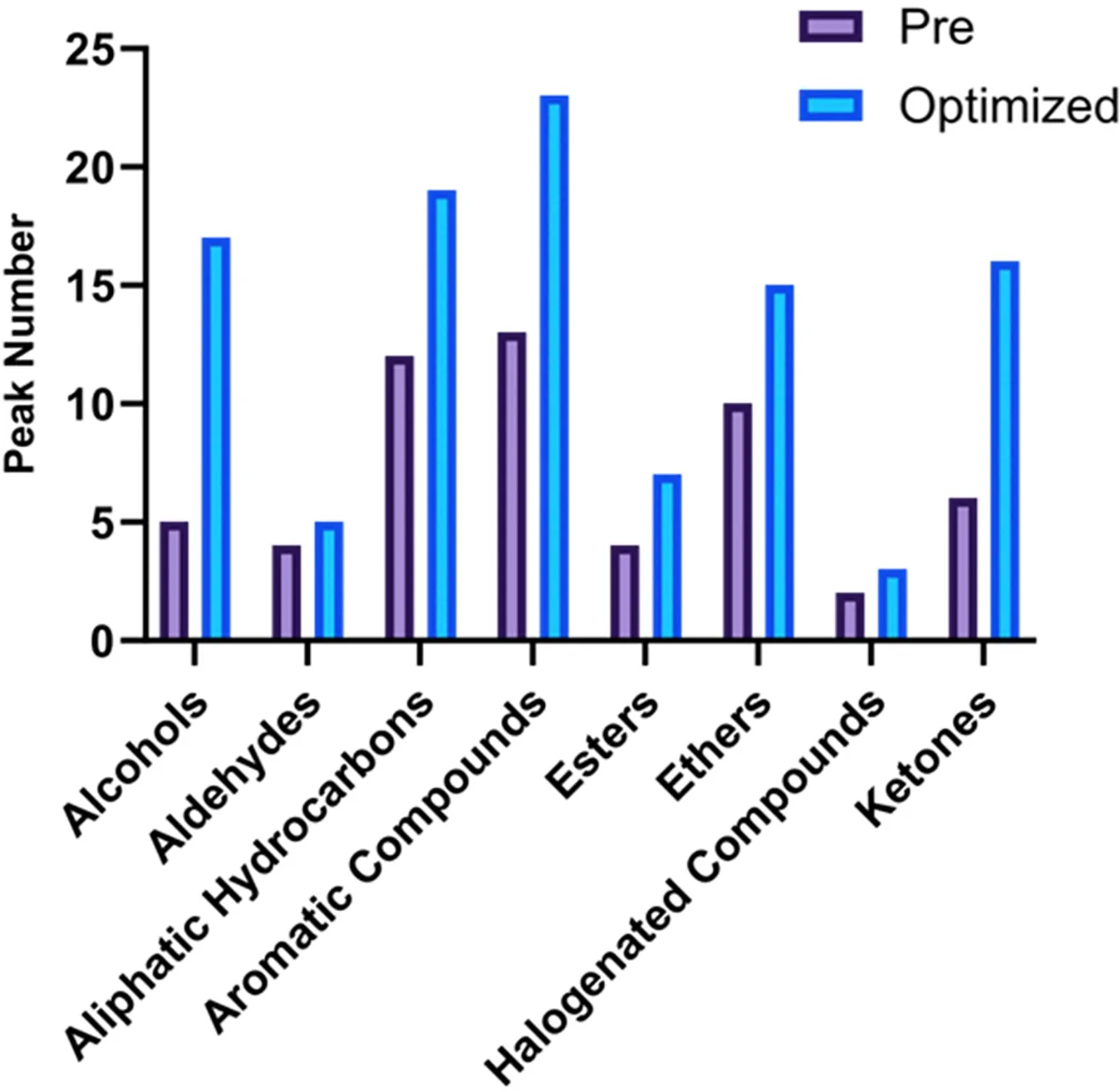
The volatile compounds that are 100% present across 10 sets of mixed BLAF samples in eight chemical classes extracted using the pre-optimization and optimized method.

In addition, we hypothesized that the optimized method would increase the number of extracted volatile compounds consistently present in BALF samples. The pre-optimization samples had 56 features consistently detected in samples, while the optimized method had 108 features consistently detected (Figure 8). We conclude that increased peak intensity can make the peak identification more consistent.

Among the 108 identified compounds, 52 compounds were presented in both methods. Fifty-six compounds were only identified with the optimized method. Only four compounds that were constantly present using the pre-optimization method were not observed in the optimized method. We observed an increase in all eight chemical classes. Alcohols and ketones showed the most significant increases, at 340% and 267%, respectively. For the other six chemical classes, the increase ranged from 125% to 177%. Therefore, the optimized parameters not only enhance the total peak area and total peak number extracted but also improve the consistency in extracting, detecting, and identifying volatile compounds.

## 4. Conclusions

This study optimized a series of HS-SPME parameters, including headspace vial size, dilution factor, extraction time, extraction temperature, and salt concentration. The effectiveness of the optimized method was validated by conducting a comparative analysis involving the extraction of ten sample sets utilizing both the pre-optimization and optimized methods. The results suggest that the optimization has increased the total peak area, total peak number, and number of compounds constantly detected in all samples. The increase was also observed across major chemical classes.

However, it is worth acknowledging the inherent challenges and limitations in this study. First, considering only the factors we have used is not sufficient for a fully comprehensive optimization. Additional parameters, such as pH, agitation mode, and agitation speed, may also be necessary to achieve more thorough results. Second, while we aim to increase the total peak area and peak numbers to benefit the untargeted screening of samples, exploring the optimum conditions for specific compounds or chemical classes is challenging. Furthermore, it is also challenging to explain the observations by existing HS-SPME theory. Thus, an authentic chemical standard addition should be employed to explore the ideal conditions for individual compounds. A more sophisticated experimental design and increased sample size are necessary to fully explain our result by theory.

## Supplementary Material

Suppl Tables

Suppl Figures

**Supplementary Materials:** The following supporting information can be downloaded at: https://www.mdpi.com/article/10.3390/separations11010027/s1. Figure S1. Pareto Chart of the response surface analysis for total peak area and total peak number. Figure S2. Perspective plots of four out of the 41 selected compounds. The unit of extraction time is minute, the unit of extraction temperature is °C. Figure S3. Relative standard deviation (RSD) of individual compound in the six samples of the center point. Table S1: Table of the known contaminants removed from the analysis. Table S2. A list of compounds selected for the optimization of extraction time and temperature, and salt concentration. Table S3. The central composite design (CCD) Minitab output table. Table S4. Reproducibility of the six samples in the center point.

## Figures and Tables

**Table 1.** HS-SPME temperature and time tested in the central composite design (CCD).

Order	Extraction Temperature (°C)	Extraction Time (Min)	Order	Extraction Temperature (°C)	Extraction Time (Min)

1	43.5	56	8	37.0	20
2	52.5	35	9	37.0	60
3	34.3	35	10	43.5	35
4	43.5	35	11	50.0	50
5	43.5	35	12	50.0	20
6	43.5	14	13	43.5	35
7	43.5	35	14	43.5	35

**Table 2.** HS-SPME parameters for the pre-optimization and optimized method.

Optimized Parameter	Pre-Optimization	Optimized

Vial Size	20 mL	10 mL
Dilution Factor	1:2 Dilution	No Dilution
Extraction Time	30 Minutes	50 Minutes
Extraction Temperature	43 °C	45 °C
Salt Addition	No Salt	40% (*w*/*v*) NaCl

## Data Availability

The data presented in this study are available within the article and its Supplementary Materials.
