# Supplementary material for: Headspace Solid-Phase Micro-Extraction Method Optimization and Evaluation for the Volatile Compound Extraction of Bronchoalveolar Lung Lavage Fluid Samples
Source: Separations. Author manuscript; Available in PMC 2026 Sep 2. (PMC13533550; doi:10.3390/separations11010027)
Supplement: Supplementary file 1 [file NIHMS2202270-supplement-Suppl_Figures.pdf]

Supplementary Material

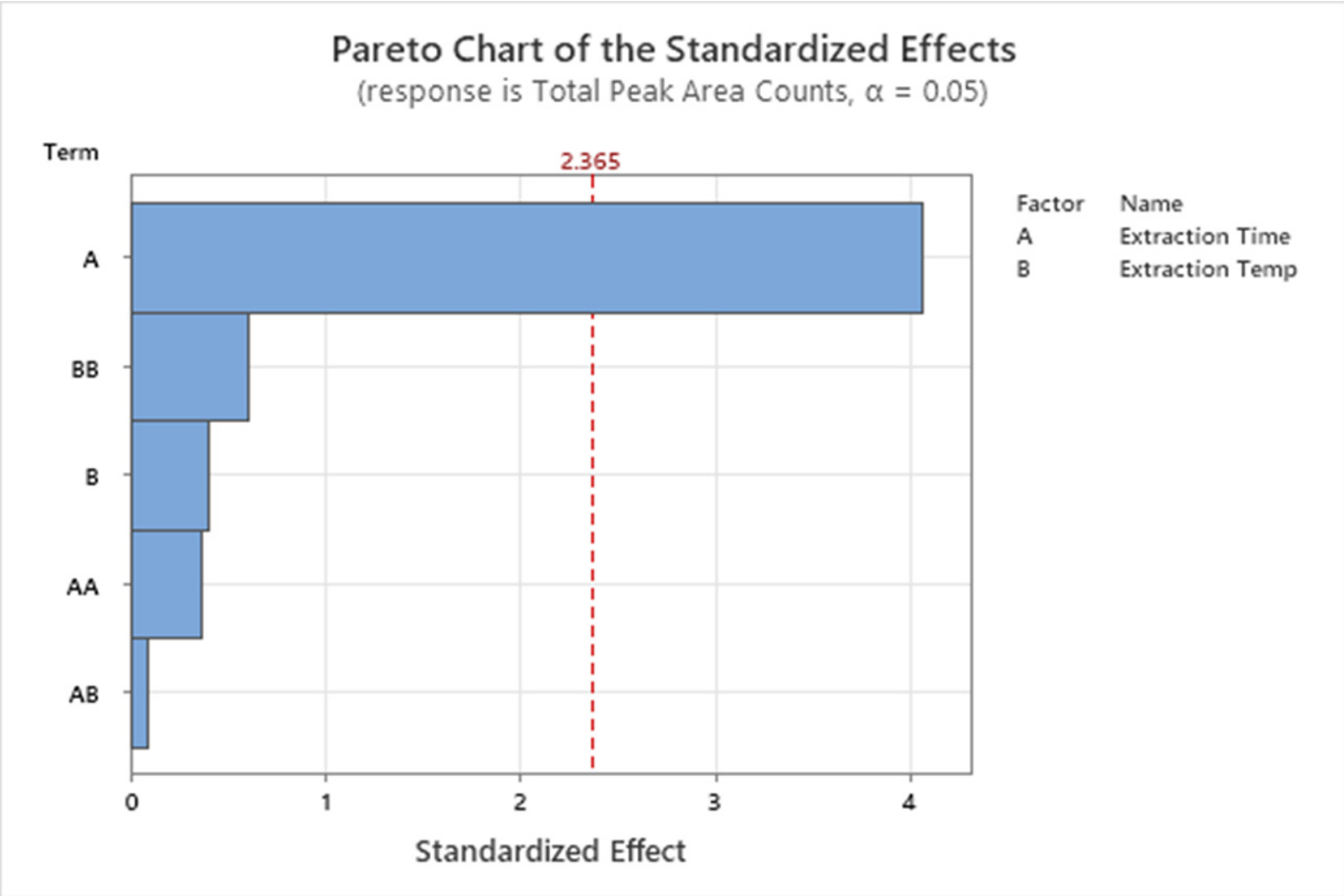

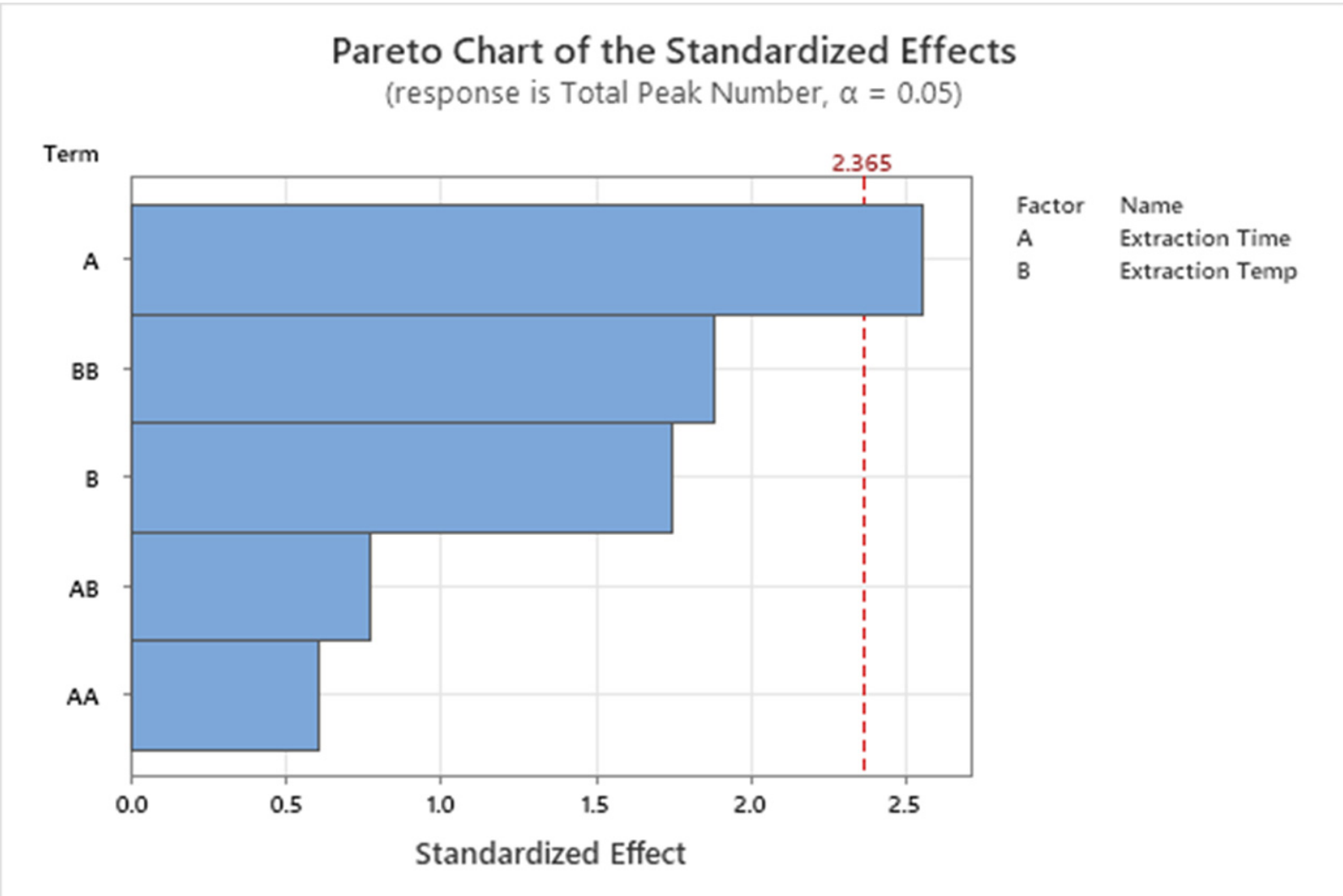

Figure S1. Pareto Chart of the response surface analysis for total peak area and total peak number.

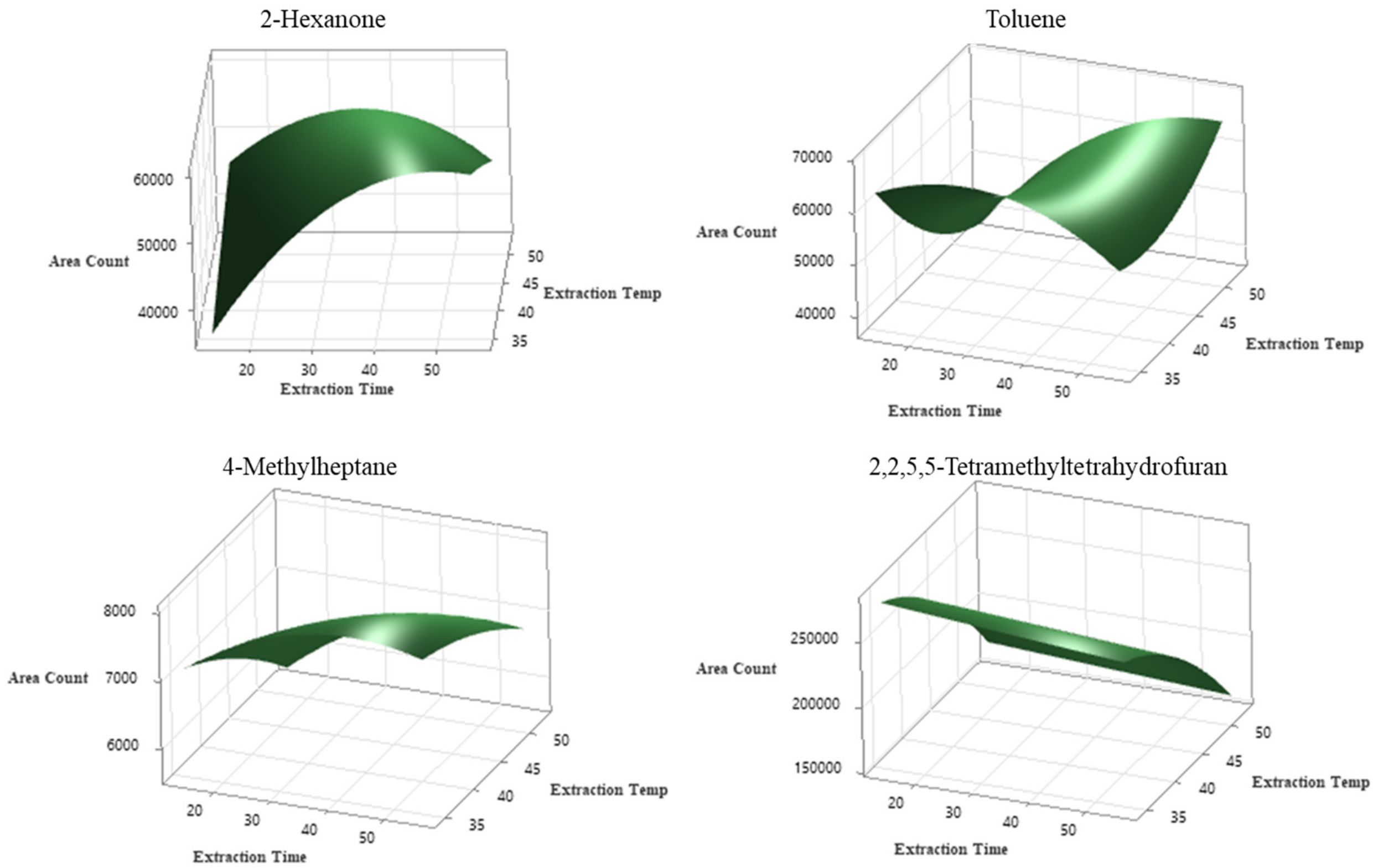

Figure S2. Perspective plots of four out of the 41 selected compounds. The unit of extraction time is minute, the unit of extraction temperature is °C.

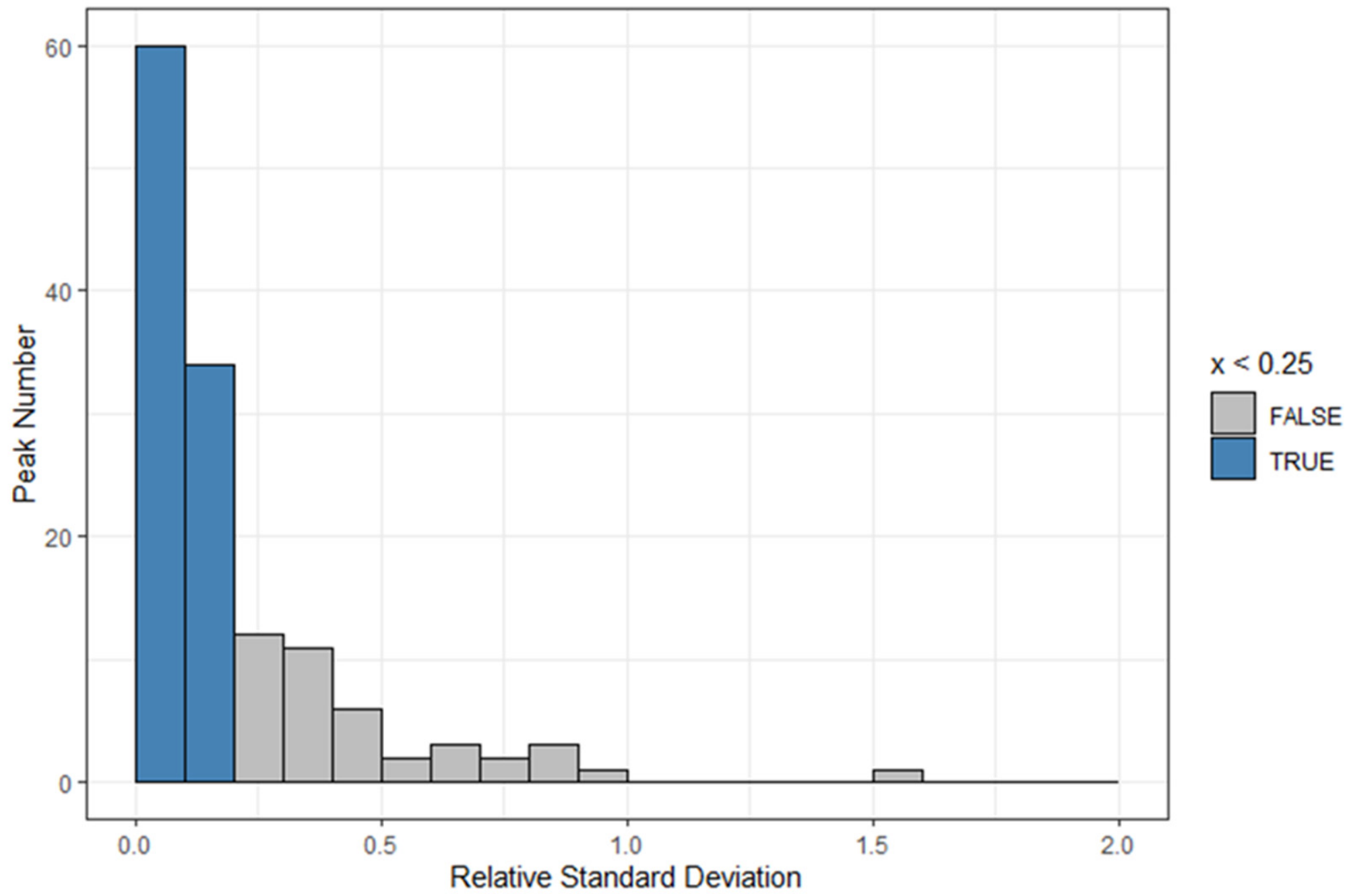

Figure S3. Relative standard deviation (RSD) of individual compound in the six samples of the center point.
